# New adhesin functions of surface-exposed pneumococcal proteins

**DOI:** 10.1186/1471-2180-10-190

**Published:** 2010-07-12

**Authors:** Cécile Frolet, Meryam Beniazza, Laure Roux, Benoit Gallet, Marjolaine Noirclerc-Savoye, Thierry Vernet, Anne Marie Di Guilmi

**Affiliations:** 1 Laboratoire d'Ingénierie des Macromolécules, Institut de Biologie Structurale (UMR 5075, Université Joseph Fourier, CNRS, CEA), Partnership for Structural Biology, Grenoble, France

## Abstract

**Background:**

*Streptococcus pneumoniae *is a widely distributed commensal Gram-positive bacteria of the upper respiratory tract. Pneumococcal colonization can progress to invasive disease, and thus become lethal, reason why antibiotics and vaccines are designed to limit the dramatic effects of the bacteria in such cases. As a consequence, pneumococcus has developed efficient antibiotic resistance, and the use of vaccines covering a limited number of serotypes such as Pneumovax^® ^and Prevnar^® ^results in the expansion of non-covered serotypes. Pneumococcal surface proteins represent challenging candidates for the development of new therapeutic targets against the bacteria. Despite the number of described virulence factors, we believe that the majority of them remain to be characterized. This is the reason why pneumococcus invasion processes are still largely unknown.

**Results:**

Availability of genome sequences facilitated the identification of pneumococcal surface proteins bearing characteristic motifs such as choline-binding proteins (Cbp) and peptidoglycan binding (LPXTG) proteins. We designed a medium throughput approach to systematically test for interactions between these pneumococcal surface proteins and host proteins (extracellular matrix proteins, circulating proteins or immunity related proteins). We cloned, expressed and purified 28 pneumococcal surface proteins. Interactions were tested in a solid phase assay, which led to the identification of 23 protein-protein interactions among which 20 are new.

**Conclusions:**

We conclude that whether peptidoglycan binding proteins do not appear to be major adhesins, most of the choline-binding proteins interact with host proteins (elastin and C reactive proteins are the major Cbp partners). These newly identified interactions open the way to a better understanding of host-pneumococcal interactions.

## Background

*Streptococcus pneumoniae *is a common bacteria of the commensal flora and together with other bacterial species, colonizes the nasopharyngeal niche and upper respiratory tract. Pneumococcal colonization is mostly asymptomatic, but can progress to respiratory or even systemic disease, causing the majority of community-acquired pneumonia and invasive diseases such as meningitis and bacteremia. Risk groups include young children, elderly people and patients with immunodeficiencies. In USA and Europe the annual incidence of invasive pneumococcal infections ranges from 10 to 100 per 100 000 with a mortality rate of 10 to 50%; the highest incidence concerns people older than 65 years [1]. The burden of pneumococcal pneumonia is very high in developing countries, and estimated to cause every year the death of more than 1 million children under the age of five. The current seven-valent conjugate vaccine for children is effective against pneumococcal invasive diseases caused by the vaccine-type strains. As more than 90 serotypes have been described, the vaccine coverage is limited and non-vaccine serotypes replacement is a serious threat for the near future [2]. The search for new vaccine candidates that would elicit protection against a broader range of pneumococcal strains or for new drugs to circumvent pneumococcal invasive disease is of tremendous interest.

Over the past 20 years, the importance of proteins for *S. pneumoniae *virulence has become clear. Research has been stimulated by the observation that pneumococcal proteins, and more precisely, surface-exposed proteins, represent promising candidates for the development of vaccines that could be common to all pneumococcal serotypes [3]. Mechanisms and pneumococcal factors that enable host epithelial and tissue barriers to be breached during the progression from colonization to invasive infection are still poorly understood. The role of the capsular polysaccharides in virulence has long been studied [4]. In order to better understand the pathogenic processes of pneumococcus, screens have been conducted, with very diverse methodologies, which allowed the identification of proteins potentially involved in host-pathogen interactions [5-9]. It now appears clearly that cell-surface proteins participate in many stages of the colonization process and/or the disease transition.

One of the first identified virulence factor of the pneumococcus is the toxin pneumolysin [10] which is able to interfere with the immune system [11,12] as well as directly destabilize host's membranes [13]. Interactions of PspA and CbpA with lactoferrin and factor H, respectively as well as proteolysis of IgA1 play important roles in the escape from the innate immune system [14-16]. The pneumococcal glycosidases NanA, NanB and SpnHL cleave terminal sugars from human glycoconjugates, which might reveal receptors for bacterial adherence and/or help for spreading of the bacteria [17]. Contrary to other pathogenic bacteria, very few interactions of pneumococcal proteins with extracellular matrix components have been described. One example is the interaction of PavA with fibronectin [18]. Direct adherence of pneumococci to epithelial cells was shown to be mediated by choline-binding protein A (CbpA) and PsaA which bind to polymeric Ig receptor and E-cadherin, respectively [19-22]. Finally, a way to progress into host tissue is to recruit the host protease plasmin at the bacterial surface. We recently demonstrated that the pneumococcal surface-exposed CbpE is a receptor for the plasminogen (as for enolase [23] and GAPDH [24]), activation of which into plasmin facilitates traversal of *S. pneumoniae *through (i) a reconstituted basement membrane, and (ii) epithelial and endothelial cell barriers via a pericellular route [25,26].

Beside the secreted or membrane-anchored protein associated with N-terminal peptide signal, three major groups of pneumococcal cell-surface proteins have been identified from specific sequence motifs which are related to three different attachment modes to the cell wall, composed by peptidoglycan, teichoic acids and lipoteichoic acids. Teichoic and lipoteichoic acids are decorated with phosphorylcholine (PCho) residues that anchor a group of proteins, the choline-binding proteins (already mentioned as Cbps). These proteins harbor repeated sequences of approximately 20 amino acids, the choline-binding module, generally present in the C-terminal part of the protein. Two to twelve modules form the choline-binding domain is attached to PCho in a non-covalent manner. Beside the choline-binding domain, the amino-acid sequences vary greatly and for some Cbps, various enzymatic activities or binding properties have been identified. The virulence factors PspA, CbpA, LytA and CbpE are part of this protein family. Secondly, in Gram-positive bacteria, proteins can be covalently linked to the peptide moiety of the peptidoglycan [27]. Transpeptidase enzymes called sortases catalyze this anchorage on a specific amino-acid sequence motif: LPXTG. This motif can vary from the canonical LPXTG sequence, this is the case for the pilin proteins (RrgA: YPRTG; RrgB: IPQTG; RrgC: VPDTG). The pneumococcal glycosidases NanA, and SpnHL are members of this LPXTG proteins family. Thirdly, cell-surface lipoproteins are covalently linked to the membrane phospholipids through the N-terminus LXXC motif recognized by the signal peptidase II. PsaA is a lipoprotein.

The availability of genomic sequence data for pneumococcal strains has facilitated the identification of additional pneumococcal surface proteins, relying on searches for specific signatures in sequences of open reading frames. For example, the initial analysis of the genomic sequence of the TIGR4 strain [28] identified 70 genes encoding for proteins predicted to be exposed at the surface of the pneumococcus, using one of the 3 attachment modes. This protein set included 19 predicted proteins with the peptidoglycan anchor LPXTG-like motif, 15 predicted Cbps, 36 proteins with putative lipid-attachment motifs (predicted lipoproteins) [28]. In the R6 strain, a comparable set of proteins display bacterial surface motifs even though not in the same number: 13 LPXTG proteins linked to the peptidoglycan, 10 Cbps and 109 lipoproteins (this number is different than in the TIGR4 strain probably because the authors used different algorithms to predict the lipoproteins). The authors mentioned that overall 471 proteins contain a predicted signal peptide sequence, an indication of their bacterial surface location, either through membrane anchoring or by secretion in the extracellular space and bound somehow to the cell wall [29].

To date, pneumococcal surface proteins acting as virulence factors and playing a role in colonization and disease are overall about 15 (mainly the ones described previously in this text). Taking into account the large number of predicted surface-exposed, and the lack of knowledge on key aspects of the physiopathology of the pneumococcus, we assume that understanding of pneumococcal disease might greatly profit from the study of yet unstudied surface-exposed proteins. In order to identify new host-pneumococcal interactions that may play roles in colonization and disease progress, we have designed a global screening strategy. We first evaluated the ability of the pneumococcus to adhere to host components. Then we cloned and expressed pneumococcal proteins from the Cbps and the LPXTG protein families to systematically test the interactions of these proteins against host proteins. We thus obtained a map of pneumococcal surface proteins interactions with twelve mammalian proteins putatively encountered during the colonization and/or invasion stages. This work allowed the identification of new protein-protein interactions between Cbp, LPXTG proteins and host proteins, and gives renewed view of the respective roles of Cbp and LPXTG proteins, opening the route for in depth study of the interactions uncovered.

## Results

### Binding of pneumococcal strains R6 and TIGR4 to host proteins

**Figure 1:**
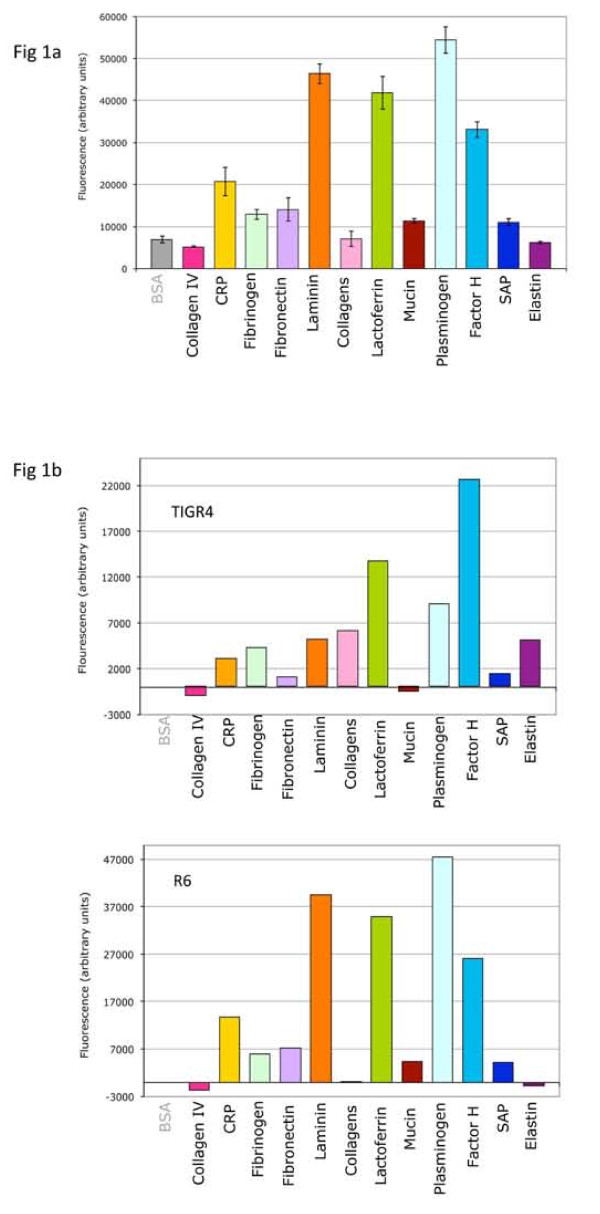
***Streptococcus pneumoniae *interaction with mammalian proteins**. FITC labeled bacteria from the R6 and TIGR4 strains were tested for their interaction with several components of the host, extracellular matrix component, circulating proteins or immunity related proteins. BSA is used as a negative control. One representative experiment is presented in each case. (a) R6 binding pattern. Error bars correspond to the standard deviation of quadruplicates within each sample. (b) Comparison of TIGR4 and R6 and binding pattern. The relative values (residual BSA blank subtracted) are presented for comprehensive comparison of the binding patterns.

We first investigated the ability of pneumococcal strains to interact with a wide range of host proteins likely encountered by bacterial pathogens [30]: extracellular matrix proteins (collagens, elastin, fibronectin, laminin, mucin), circulating plasma proteins acting in the coagulation cascade (fibrinogen, plasminogen) and proteins involved in the innate immune defense (lactoferrin, CRP, SAP, factor H). Binding of the R6 strain to these host proteins was tested in a solid-phase assay. Host proteins or Bovine Serum Albumine (BSA) as a negative control were coated on a multi-well plate. FITC-labeled pneumococcus was added and FITC signal was measured after washings of the plate to compare binding of the pneumococcus to BSA and host components (Fig. 1a). The threshold for considering a positive interaction was twice the BSA negative control. Consequently, no significant binding of R6 bacteria was detected to collagen type IV, to a mix of different collagens or to elastin. A low binding level (two to three times above the BSA binding level) was observed for CRP, fibrinogen, fibronectin, mucin and SAP while a higher level of binding was detected to laminin, lactoferrin, plasminogen and factor H (Fig. 1a). A similar experiment has been performed with the encapsulated TIGR4 strain (Fig 1b). No, or very low binding level, was observed for the TIGR4 strain to the collagen type IV, fibronectin, mucin and SAP and a slight higher interaction with CRP, fibrinogen, laminin, collagens and elastin. A high binding level of the TIGR4 strain was measured to lactoferrin, plasminogen and factor H (Fig 1b). Both R6 and TIGR4 strains bind strongly to the lactoferrin and factor H, while the high binding level of R6 to laminin and plasminogen is less important in the case of the TIGR4 strain, the latter harbors a higher recognition property to the elastin compared to the R6 strain.

Interaction of pneumococcal cells with laminin [31], CRP [32], fibronectin [33] and mucin [34] have been described in the literature. All other identified interactions are not described to date, and to investigate these interactions at the molecular level, we designed an approach to systematically test interactions between selected pneumococcal surface proteins and host proteins.

### Identification, expression and purification of choline-binding proteins (Cbps)

**Figure 2:**
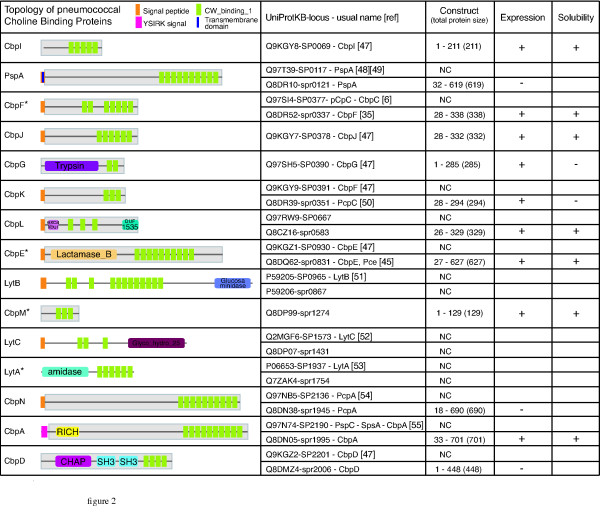
***Streptococcus pneumoniae *Choline-binding proteins**. Topology of the Cbps was analyzed on R6 proteins when existing otherwise TIGR4 by SMART search of PFAM domains http://smart.embl-heidelberg.de/. Resulting general topology of the protein is figured, domains are named with PFAM nomenclature. YSIRK stands for the Gram-positive signal peptide (Pfam entry: PF04650). * refers to proteins for which the number of choline-binding repeats has been determined by crystallography, and was thus used in the table [36,45-47]. The cloned part of the protein is included in the grey box. Protein and locus nomenclature together with the common names of the proteins, and references for their original discovery are listed in the second column. The third column figures the construct boundaries, and size of the complete protein, NC: Not Cloned. The latter columns display the positive or negative results of expression and solubility of the corresponding proteins.

We built a list of the Cbps present in the R6 and TIGR4 genomes using the published data [28,29]. From these sequences, 10 genes encoding Cbps were predicted in the R6 genome, and 15 in the TIGR4 genome (Fig 2). We systematically compared the TIGR4 and R6 protein databases derived from their complete genome sequence in order to get a list of orthologs between the two organisms. This work was facilitated by the high level of conservation of gene organization between both genomes. This analysis led to the identification of two new Cbps in the R6 genome not identified in the initial study [29], namely spr0583 and spr1274 (Fig 2). In order to homogenize the nomenclature, we named these newly identified choline-binding proteins CbpL (encoded by spr0583 and SP0667 in the R6 and TIGR4 strains, respectively) and CbpM (encoded by spr1274 in R6, the TIGR4 SP1417 locus being a pseudo-gene). The CbpG [35] (SP0390) ortholog in the R6 strain is split in two proteins: spr0349 contains a peptidase domain and spr0350 is a very small protein (42 aa) with a single predicted choline-binding domain. Thus, CbpG does not seem to exist in the R6 strain as a Cbp. Taking all these data together, we conclude that the R6 and TIGR4 genomes encode for 12 and 14 Cbps respectively. Figure 2 gives a comprehensive overview of the Cbps in *Streptococcus pneumoniae *strains R6 and TIGR4. This classification points out that names previously used to identify the Cbps were confusing. For instance, the ortholog of PcpC in TIGR4 (SP0377) is named CbpF in R6 (spr0337) and the ortholog of CbpF in TIGR4 (SP0391) is PcpC in R6 (spr0351). As CbpF was studied in R6 [36] under that name, we chose to rename SP0391 and spr0351 CbpK. PcpA was also renamed CbpN. We didn't rename well studied Cbps such as PspA, LytA, LytB and LytC. A similar analysis has been performed with the strains G54 (serotype 19F) and Hungary 19A-6 (serotype 19A) (Table S1). The G54 strain contains 14 Cbps among which only the CbpJ is absent, while 12 Cbps have been identified in the Hungary 19A-6 strain which does not express CbpI, CbpJ and CbpG.

The level of sequence identity between the R6 and TIGR4 Cbps orthologs was determined by Kalign http://msa.sbc.su.se/cgi-bin/msa.cgi and ranged between 84% and 99%, except for PspA with 63% of sequence identity. Some of the Cbps present slight differences in their general topology: TIGR4 CbpK is larger than R6's and has 3 more choline-binding domains. TIGR4 CbpN is reduced by 3 choline-binding domains. Both CbpA have roughly the same size, but 2 more choline-binding domains are predicted in the R6 protein.

Cbps can be separated into three classes: some of them have no predicted domain except the choline-binding domain, as CbpI, PspA, CbpF, CbpJ, CbpK, CbpM (which is the shortest with 129 aa), and CbpN (which is the longest with 690 aa and 10 choline-binding domains). Other Cbps present additional domains with identified enzymatic functions (CbpG, CbpE, Lyt proteins). Finally some Cbps exhibit additional predicted domains of unknown functions (CbpL, CbpA, CbpD). All the genes encoding the Cbps were cloned, excluding genes coding for the Lyt proteins as their roles are well documented. CbpE was already cloned in the laboratory [25]. PspA, CbpN and CbpD were not expressed. CbpG and CbpK were expressed as an insoluble form: these proteins were not studied further. CbpA, CbpE, CbpF, CbpI, CbpJ, CbpL and CbpM were successfully purified.

### Expression and purification of LPXTG proteins

**Figure 3:**
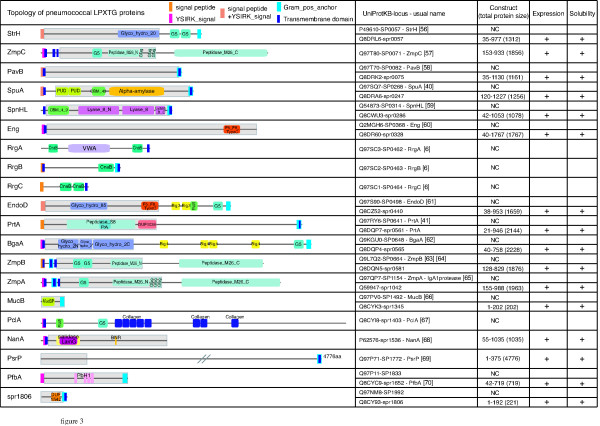
***Streptococcus pneumoniae *LPXTG proteins**. Topology of the LPXTG proteins was analyzed on R6 proteins when existing otherwise TIGR4 by SMART search of PFAM domains http://smart.embl-heidelberg.de/. Resulting general topology of the protein is figured, domains are named with PFAM nomenclature. YSIRK stands for the Gram-positive signal peptide (Pfam entry: PF04650). The cloned part of the protein is included in the grey box. The second column gives the protein and locus nomenclature together with the common names of the proteins, and references for their original discovery. The third column figures the construct boundaries, and size of the complete protein, NC: Not Cloned. The latter columns bring out that every cloned genes gave soluble proteins produced.

A comparable analysis has been conducted with the LPXTG proteins (Fig 3). There are genes for 19 and 13 LPXTG family members identified in the TIGR4 and R6 genomes, respectively [28,29]. Ten LPXTG proteins are common to the R6 and TIGR4 genomes meaning that some of these surface-exposed proteins are specific to either R6 or TIGR4 strains. Five LPXTG proteins are specific of TIGR4, among which the pilin proteins encoded at loci SP0462, SP0463 and SP0464 and thought to be covalently associated to each other via their LPXTG-like motif by specific pilus-sortase enzymes [37]. Because these particular LPXTG proteins are not linked to the peptidoglycan by the housekeeping sortase A, they have not been included in this study. Two other LPXTG proteins are present in the TIGR4 strain and absent from the R6 strain: the metalloprotease ZmpC and PsrP, a very large protein (4776 aa) essentially composed of a serine rich region [38]. Three new R6 orthologs were identified: proteins EndoD (SP0498 = spr0440), ZmpB (SP0664 = spr0581) and ZmpA (SP1154 = spr1042) (Fig 3). NanA (spr1536) and PclA (= spr1403) are present in the R6 strain but not in TIGR4. Among the LPXTG proteins, spr0400 does not have a LPXTG motif, as was initially reported [29] nor a Gram-positive anchor, was thus excluded from our study. CbpA (SP2190) is identified both as Cbp and LPXTG protein in the TIGR4 annotations. As we did not find a LPXTG motif in SP2190, it was excluded from the LPXTG proteins list and kept with the Cbps (Fig 2 &3). The initial inaccurate annotation as an LPXTG protein likely originates from the presence of an allelic variant of CbpA harboring an LPXTG motif in some pneumococcal strains [15,39]. Finally, the R6 strain has 15 genes encoding for LPXTG proteins compared to 18 for the TIGR4 strain. Protein sizes range from 202 aa (MucB) to 4776 aa (PsrP). Some of them are enzymes (Fig 3) while others may be involved in molecular recognition (SpuA and SpnHL harbor carbohydrate binding modules...). The sequence identity between LPXTG orthologs found in R6 and TIGR4 strains ranged between 89% and 100%, except for the ZmpB protein which sequence identity is 52%. Most of the LPXTG proteins have a comparable domain organization in both strains. A similar analysis has been performed with the strains G54 and Hungary 19A-6 (Table S2). The G54 and Hungary 19A-6 strains encode for 15 and 18 LPXTG proteins, respectively. The pilus operon is missing in the G54 strain as well as the PclA and PsrP sequences, neither the genes encoding for ZmpC nor PclA are present in the Hungary 19A-6 strain.

As LPXTG proteins are often large, selected domains were cloned for protein expression for most of them (Fig 3). All cloning were successful except for PclA. All the constructs were positively tested for protein expression and led to the production of soluble recombinant forms.

### Protein interactions screening by solid-phase assay

In order to study on a large scale the interactions of the pneumococcal choline-binding proteins and LPXTG proteins with host components, a solid-phase test to screen for interactions between the purified His-tagged pneumococcal proteins and host components was designed and automated. Chosen mammalian proteins, already tested with pneumococci (Fig. 1), were either part of the extracellular matrix (collagens, fibronectin, laminin, mucin, elastin) or circulating proteins (CRP, lactoferrin, fibrinogen, plasminogen, factor H, SAP). These proteins were coated on a 96 wells plate and the interaction with the purified recombinant His-tagged pneumococcal proteins was detected using an anti His-Tag antibody coupled to the HRP enzyme and revealed by chemiluminescence. Each interaction experiment was conducted at least three times using two or more different protein preparations. Interactions observed in a majority of at least three independent experiments are considered as positives (Table 1).

**Table 1:** Interactions between host and pneumococcal proteins

	Collagens	Collagen IV	Fibrinogen	Laminin	Elastin	CRP	Factor H	Plasminogen	Fibronectin Mucin Lactoferrin
CbpA							**+**		

CbpI					**+**	**+**			

CbpM						**+**			

CbpJ						**+**			

CbpL	**+**				**+**	**+**			

CbpF					**+**			**+**	

CbpE			**+**	**+**			**+**	**+**	

StrH									

ZmpC									

SpuA								**+**	

SpnHL									

Eng								**+**	

EndoD									

PrtA		**+**						**+**	

BgaA									

ZmpB		**+**							

NanA	**+**	**+**	**+**						

PfbA									

spr1806								**+**	

This table summarizes the ELISA interaction experiments. Each positive interaction was validated in a majority of at least 3 independent experiments (see material and methods) and is represented by a cross. Empty boxes stand for an absence of detected interaction. Pneumococcal proteins are figured on the left and the tested mammalian proteins are at the top of the table, those giving no interaction have been grouped at the right of the table.

#### Interaction profile of the choline-binding proteins

Elastin is the extracellular matrix component showing the largest number of interactions with Cbps: CbpI, CbpL and CbpF, while collagens interact only with CbpL and laminin only with CbpE (Table 1). The most frequent interactions have been observed with circulating proteins, such as CRP, factor H and plasminogen. Four different Cbps interact with CRP: CbpI, CbpM, CbpJ and CbpL. CbpE and CbpA, interact with factor H, the latter interaction confirming previous results [40], Plasminogen interacts with CbpE and CbpF (Table 1). Interactions between CbpE and laminin or plasminogen confirm our previous observations to which we add factor H herein [25].

#### Interaction profile of the LPXTG proteins

Even though all expressed LPXTG proteins were produced as soluble recombinant proteins, some of them gave poor purification yield or poor signal detection during the screen. These restrictions led to the abandon in the screen assay of PavB, ZmpA, MucB and PsrP. The most common interactions encountered with the LPXTG candidates involved the collagen IV (PrtA, ZmpB, NanA and spr1806) and the plasminogen (SpuA, Eng, PrtA and spr1806) (Table 1). NanA also interacts with collagens and fibrinogen (Table 1). The interaction level of NanA with lactoferrin was not significant in our assay contrary to a previous observation [17].

### Dose-responses curves

**Figure 4:**
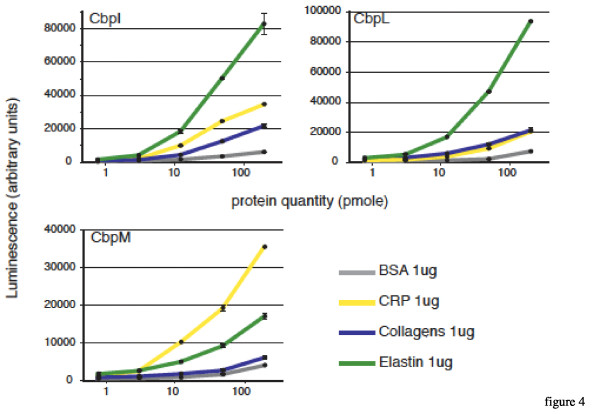
**Dose dependent binding of chosen Cbps to CRP, elastin and collagens**. Increasing concentrations of His-Tagged Cbps (from 0,8 to 200 pmole) have been bound to 1 μg of BSA as a control, CRP, collagens and elastin. The quantity of bound protein is detected in a luminometer using an HRP conjugated antibody directed against the His-Tag.

We chose to investigate the dose-response of three unstudied Cbps for which we observed host-protein binding functions: the solid-phase assay screening led to the observation that CbpL interacts with collagens, elastin and CRP, CbpI binds to elastin and CRP and CbpM binds only to CRP. In this experiment, 1 μg of each mammalian protein is coated and increasing amounts of pneumococcal proteins is used, from 0.8 to 200 pmoles per well. For all three analyzed Cbps, the interaction with mammalian proteins is dose-dependent (Fig 4). The highest level of binding of CbpL is observed with elastin, intermediate response with collagens and CRP compared with the BSA negative control (Fig 4). These data confirm the results of the screen, and also comfort the "semi-quantitative" informations about the level of binding that we obtained from the screen. The interaction of CbpI with elastin and CRP yielded the most important response levels in the dose-response measurements, in accordance with the screen assay but a significant level of interaction was also observed with collagens (Fig 4). Even though the sole interaction of CbpM which came out from the screen procedure was with CRP, confirmed in the dose-response analysis, this more detailed characterization allows to propose that CbpM interacts with elastin but too weakly to be considered as positive during the screen procedure (Fig 4). All together these results validate the procedure that we used to select the interactions that emerge from the screen.

## Discussion

We have presented an experimental set up that allowed the analysis of the binding properties of 19 surface-exposed pneumococcal proteins, leading to the screen of more than 200 interactions, most of which have never been reported in the literature before. The validity of this approach is strengthened by the fact that known interactions were « rediscovered ». For example, we confirmed the interaction between CbpA and Factor H [40]. Complementary ELISA analysis gave a confirmation of the validity of our procedure on chosen protein-protein interactions. From this screen, we conclude that whereas LPXTG proteins do not appear to be major adhesins, Cbps seem to be more important players in the adhesion processes. One explanation can be that most of the Cbps are not associated with enzymatic functions (except the Lyt proteins, CbpD, CbpE and CbpG, see Fig 2). Probably the main function of the Cbps (except for the Lyt proteins) resides in the host-pathogen interaction, and adhesion processes. Most of the LPXTG proteins do exhibit complex 'multi'-functions (enzymatic domains plus different binding domains, see Fig 3), rendering plausible the hypothesis that they have more diverse functions at the surface of the bacteria. Indeed, the results obtained tend to minimize their roles in the adhesion processes. However one has to keep in mind that often only part of the LPXTG proteins was tested as they are usually larger proteins than the Cbps. It's possible that this bias led us to miss significant interactions. Another point is that only protein-protein interactions were tested during the course of the screen. Yet carbohydrates are important components of the host, they were not included in that study and could be an important target of the LPXTG proteins, in particular for the ones that bear carbohydrate-binding modules as it was recently proven for SpuA [41]. Finally, this screen addressed a small fraction of host factors potentially involved in the interactions with the pneumococcus. Thus our screen gives an overview of some protein-protein interactions and extension on this work would require higher throughput techniques such as those based on chips. It's interesting to note that some of the LPXTG found to be adhesins during the course of this screen are proteases such as PrtA and ZmpB. One tempting hypothesis that has already been proposed for PrtA [42] could be that these proteins are involved in the cleavage of host proteins in order to penetrate into the tissues or escape the immune system. Future research will have to elucidate these questions and in particular, the fate of the mammalian proteins after the interactions.

During the course of the screen, we identified 3 Cbps, CbpI, CbpL and CbpM that interact with elastin. To the best of our knowledge, this is the first time that interactions of pneumococcal proteins with elastin are discovered. Elastin is a major component of the lungs and blood vessels, and is thus probably frequently encountered by the bacteria. CbpI and CbpL are only expressed in the TIGR4 strain and harbor a high level binding to elastin, while CbpM is specific of the R6 strain and binds weakly to elastin. These data are in accordance with the bacterial binding pattern to elastin: no interaction of the R6 strain was observed with elastin while the TIGR4 strain presents a significant binding property to elastin, indicating that in this latter strain, and despite the presence of the capsule, the recognition to elastin might be due to CbpI and CbpL (Fig. 1). These newly characterized interactions open the way to a better understanding of the contribution of choline-binding proteins during the invasion process. Considering the general interest in the identification and validation of new protein vaccine candidates, that would elicit protection against a broader range of pneumococcal strains and/or play a significant role in the virulence process, it is interesting to note that all the identified recombinant proteins that positively interact with the host proteins are also present in the G54 and Hungary 19A-6 strains, except CbpJ in both strains and CbpI in the latter strain.

We also observed an interaction between some Cbps and the CRP. The interaction between *Streptococcus pneumoniae *and CRP is one of the first identified host-pathogen interaction at the molecular level [32]. CRP stands for C Reactive Protein, with C standing for C polysaccharide, which contains the teichoic and lipoteichoic acids from pneumococcus. In fact, CRP is interacting with phosphocholines (PCho) [43] harbored by teichoic and lipoteichoic acids. The possibility exists that Cbps could harbor in their choline-binding domains enough PCho to reproduce this interaction. However, it's important to note that not every purified Cbp did interact with CRP, leaving opened the question of a direct interaction between Cbps and CRP.

## Conclusions

We have presented an experimental design that allowed the analysis of the binding properties of 19 surface-exposed pneumococcal proteins, leading to the discovery of 20 new interactions with host proteins. This screen opens the route for in depth study of the role of these surface exposed proteins in the virulence processes,

## Methods

### Cloning of the cbp and lpxtg genes

Oligonucleotides were designed to amplify the required fragments either on R6 or TIGR4 genomic DNA (ATCC BAA334D). R6 genes were preferentially cloned when existing. In order to maximize chances to get soluble proteins expressed in *E. coli *cytoplasm, we systematically eliminated the predicted signal peptides, transmembrane domains or Gram-positive anchor when present, as for CbpA (Fig 2). The Ligation Independent Cloning (LIC) technique was chosen in order to facilitate high throughput cloning steps [44]); LIC extensions were in consequence included in the primers. PCR amplification was performed using the Phusion polymerase (Finnzyme, #F530L). The amplified gene fragments were cloned into pLIM01 or pLIM12 LIC-vectors (PX'Therapeutics, Grenoble) leading to N-terminal His-Tag fusion proteins. Plasmids were transformed into *E. coli *DH5a and inserts were sequenced to verify the absence of undesired mutations (Cogenics, Grenoble). The *E. coli *strain BL21CodonPlus^®^(DE3)RIL (Stratagene #230245) was used for protein expression.

### Protein expression and purification

Transformed bacteria were precultured (3 mL) in Terrific Broth (TB) with the appropriate antibiotic, chloramphenicol 34 μg/mL, ampicillin 100 μg/mL (pLIM01 vector) or kanamycin 50 μg/mL (pLIM12 vector) at 37°C for overnight incubation. A volume of 250 mL of TB media (plus ampicillin or kanamycin only) was inoculated with the overnight culture and the bacterial growth was performed at 37°C until an OD at 600 nm of 2 was reached. The protein expression was induced by 1 mM IPTG and the culture incubation was carried on at 15°C for about 18 hours.

Bacterial culture was spun down and the pellet resuspended in an appropriate buffer composed of 50 mM Hepes pH7.0 or 50 mM Tris pH8.0 (depending on the pI of the expressed protein), 150 mM NaCl, 40 mM Imidazole and a cocktail of protease inhibitors (complete EDTA free, Roche). After cell lysis by sonication, the recombinant proteins were recovered from the soluble fraction and loaded onto a 1 ml - prepacked HisTrap™ HP (17-5247-01, GE Healthcare) column or HIS-Select^® ^High Flow Cartridge (Sigma #H7788). Column equilibration was performed in the same buffer as lysis. After extensive washing, recombinant proteins were eluted with a 20 - 500 mM imidazole gradient. The eluted fractions were analyzed on an SDS acrylamide denaturing gel. If necessary (generally when the purity of the protein appeared to be less than 90% on the gel), the purification process was continued with an ion exchange column and/or a size exclusion chromatography. Protein concentrations were determined from the absorbance at 280 nm with a spectrophotometer (Nanovue, GE healthcare). For the choline-binding proteins, yields ranged between 5 mg/liter (CbpF) and 120 mg/liter (CbpM, CbpJ) of *E. coli *culture with a purity estimated on SDS-PAGE greater than 90%. Cbps are often more stable when stored in the elution buffer of the affinity column than in PBS. The purification yields of LPXTG proteins ranged between less than 1 mg to 60 mg/liter of *E. coli *culture, with a purity level estimated on SDS-PAGE of a minimum of 75%.

### *S. pneumoniae *interactions screening by solid-phase assay

Black 96 well plates (Greiner 655077) were coated overnight at 4°C with 1 μg (in 100 μL PBS pH7.0) of the following mammalian proteins: collagen IV (Sigma, C5533), collagens (Merck, 234112), elastin (Merck, 324751), fibronectin (Merck, 341635), laminin (Sigma, L2020), fibrinogen (Sigma, F3879), mucin (Sigma, M3895), plasminogen (Sigma, P7999), lactoferrin (Sigma, L3770), C-reactive protein (Merck, 236608), serum amyloïd P component (SAP, Merck, 565190), factor H (Merck, 341274), and bovine serum albumin (BSA, Promega R3961) as a control. The plate is saturated the day after at room temperature for 1 h with 1% BSA (Sigma, A7030).

*Streptococcus pneumoniae *from the R6 strain was cultured in Todd Hewitt broth (BD) to an OD of 0.3, harvested and washed in PBS. One mg of FITC (Sigma, F7250) was diluted in 1 mL of PBS, centrifuged and the supernatant was used to resuspend the R6 pellet. The bacteria were kept 20 minutes in the dark. Afterwards, several centrifugation steps (usually 5 or 6, 4000 g-2 min) are conducted in PBS in order to remove free FITC. FITC-labelled bacteria (10^8 ^cfu) were then deposited in each well (in 50 μL of PBS, BSA 0,2%). The bacteria were left to interact for 2 h at 37°C, before washing eight times with 100 μL of PBS. The fluorescence signal was read in a fluorimeter (FLUOstar Optima, BMG Labtech).

### Protein interactions screening by solid-phase assay

White 96 well plates (Greiner 655074) were coated overnight at 4°C with 1 μg (in 100 μL PBS pH7.0) of the same mammalian proteins as in the previously described experiment: collagen IV, collagens, elastin, fibronectin, laminin, fibrinogen, mucin, plasminogen, lactoferrin, CRP, SAP, factor H, and BSA as a control. The following steps were conducted at room temperature in a Microstar^® ^lab robot (Hamilton). Saturation was performed for 1 h with 200 μL of PBS 2% BSA (Sigma, A7030). His-Tagged recombinant pneumococcal surface protein (200 pmole in 100 μL PBS) were added to each well and left for two hours, three washing steps of ten minutes in 200 μL PBS, Tween 0,03% were then performed. The anti His-HRP-coupled antibody (Sigma, A7058) was diluted 1000× in PBS Tween 0,03% BSA 0,2% and 100 μL were added to the wells. Three washings in 200 μL PBS, Tween 0,03%, followed this last step. The antibody signal was revealed with 100 μL of ECL (Pierce, 32106) and the luminescence immediately read in a FLUOstar OPTIMA (BMG Labtech). Each well was triplicated. The threshold for considering a positive interaction was twice the BSA negative control.

In order to get the maximal accuracy in the interpretation of the results, we built a specific protocol for a global analysis of the results and retained as positive the interactions observed in the majority of data set, provided from at least 3 independent experiments.

### Dose response curves

Similar protocol was used except that increasing quantities of pneumococcal His-tagged proteins were used in the interaction steps, from 0.8 to 200 pmoles. Dose-response curves are in consequence presented with a logarithmic scale.

## Authors' contributions

CF participated in the design of the study, carried out and analyzed all the experiments. The Robiomol platform (BG and MNS) participated in the gene cloning procedures. BG conceived the program for the Hamilton robot. MB and LR participated in protein purification and ELISA experiments. AMDG and CF conceived the study; AMDG and TV coordinated the study; CF, AMDG and TV drafted the manuscript. All authors read and approved the final manuscript.

## Supplementary Material

Additional file 1 **Choline-Binding Proteins in R6, TIGR4, G54 and Hungary 19A-6**. Click here for file

Additional file 2 **LPxTG Proteins in R6, TIGR4, G54 and Hungary 19A-6**. Click here for file

## References

[B1] Cartwright K Pneumococcal disease in western Europe: burden of disease, antibiotic resistance and management Eur J Pediatr 2002 161 4 188 195 10.1007/s00431-001-0907-3 12014384

[B2] Cohen R Levy C Bonnet E Grondin S Desvignes V Lecuyer A Fritzell B Varon E Dynamic of pneumococcal nasopharyngeal carriage in children with acute otitis media following PCV7 introduction in France Vaccine 2009 Available online 31 May 2009 10.1016/j.vaccine.2009.05.037 19490958

[B3] Giefing C Meinke AL Hanner M Henics T Bui MD Gelbmann D Lundberg U Senn BM Schunn M Habel A Discovery of a novel class of highly conserved vaccine antigens using genomic scale antigenic fingerprinting of pneumococcus with human antibodies J Exp Med 2008 205 1 117 131 10.1084/jem.20071168 18166586 PMC2234372

[B4] MacLeod CM Kraus MR Relation of virulence of pneumococcal strains for mice to the quantity of capsular polysaccharide formed in vitro J Exp Med 1950 92 1 1 9 10.1084/jem.92.1.1 15422092 PMC2136027

[B5] Zysk G Bongaerts RJ ten Thoren E Bethe G Hakenbeck R Heinz HP Detection of 23 immunogenic pneumococcal proteins using convalescent-phase serum Infect Immun 2000 68 6 3740 3743 10.1128/IAI.68.6.3740-3743.2000 10816539 PMC97670

[B6] Hava DL Camilli A Large-scale identification of serotype 4 *Streptococcus pneumoniae *virulence factors Mol Microbiol 2002 45 5 1389 1406 12207705 PMC2788772

[B7] Polissi A Pontiggia A Feger G Altieri M Mottl H Ferrari L Simon D Large-scale identification of virulence genes from Streptococcus pneumoniae Infect Immun 1998 66 12 5620 5629 9826334 10.1128/iai.66.12.5620-5629.1998 PMC108710

[B8] Wizemann TM Heinrichs JH Adamou JE Erwin AL Kunsch C Choi GH Barash SC Rosen CA Masure HR Tuomanen E Use of a whole genome approach to identify vaccine molecules affording protection against Streptococcus pneumoniae infection Infect Immun 2001 69 3 1593 1598 10.1128/IAI.69.3.1593-1598.2001 11179332 PMC98061

[B9] Rigden DJ Galperin MY Jedrzejas MJ Analysis of structure and function of putative surface-exposed proteins encoded in the Streptococcus pneumoniae genome: a bioinformatics-based approach to vaccine and drug design Crit Rev Biochem Mol Biol 2003 38 2 143 168 10.1080/713609215 12749697

[B10] Libman E A pneumococcus producing a peculiar form of hemolysis Proc NY Pathol Soc 1905 5

[B11] Paton JC Ferrante A Inhibition of human polymorphonuclear leukocyte respiratory burst, bactericidal activity, and migration by pneumolysin Infect Immun 1983 41 3 1212 1216 6885160 10.1128/iai.41.3.1212-1216.1983 PMC264628

[B12] Paton JC Rowan-Kelly B Ferrante A Activation of human complement by the pneumococcal toxin pneumolysin Infect Immun 1984 43 3 1085 1087 6698602 10.1128/iai.43.3.1085-1087.1984 PMC264298

[B13] Boulnois GJ Paton JC Mitchell TJ Andrew PW Structure and function of pneumolysin, the multifunctional, thiol-activated toxin of Streptococcus pneumoniae Mol Microbiol 1991 5 11 2611 2616 10.1111/j.1365-2958.1991.tb01969.x 1779752

[B14] Hammerschmidt S Bethe G Remane PH Chhatwal GS Identification of pneumococcal surface protein A as a lactoferrin-binding protein of Streptococcus pneumoniae Infect Immun 1999 67 4 1683 1687 10085004 10.1128/iai.67.4.1683-1687.1999 PMC96514

[B15] Janulczyk R Iannelli F Sjoholm AG Pozzi G Bjorck L Hic, a novel surface protein of Streptococcus pneumoniae that interferes with complement function J Biol Chem 2000 275 47 37257 37263 10.1074/jbc.M004572200 10967103

[B16] Romanello V Marcacci M Dal Molin F Moschioni M Censini S Covacci A Baritussio AG Montecucco C Tonello F Cloning, expression, purification, and characterization of Streptococcus pneumoniae IgA1 protease Protein Expr Purif 2006 45 1 142 149 10.1016/j.pep.2005.07.015 16146695

[B17] King SJ Hippe KR Gould JM Bae D Peterson S Cline RT Fasching C Janoff EN Weiser JN Phase variable desialylation of host proteins that bind to Streptococcus pneumoniae in vivo and protect the airway Mol Microbiol 2004 54 1 159 171 10.1111/j.1365-2958.2004.04252.x 15458413

[B18] Holmes AR McNab R Millsap KW Rohde M Hammerschmidt S Mawdsley JL Jenkinson HF The pavA gene of Streptococcus pneumoniae encodes a fibronectin-binding protein that is essential for virulence Mol Microbiol 2001 41 6 1395 1408 10.1046/j.1365-2958.2001.02610.x 11580843

[B19] Zhang JR Mostov KE Lamm ME Nanno M Shimida S Ohwaki M Tuomanen E The polymeric immunoglobulin receptor translocates pneumococci across human nasopharyngeal epithelial cells Cell 2000 102 6 827 837 10.1016/S0092-8674(00)00071-4 11030626

[B20] Anderton JM Rajam G Romero-Steiner S Summer S Kowalczyk AP Carlone GM Sampson JS Ades EW E-cadherin is a receptor for the common protein pneumococcal surface adhesin A (PsaA) of Streptococcus pneumoniae Microb Pathog 2007 42 5-6 225 236 10.1016/j.micpath.2007.02.003 17412553

[B21] Lu L Ma Y Zhang JR Streptococcus pneumoniae recruits complement factor H through the amino terminus of CbpA J Biol Chem 2006 281 22 15464 15474 10.1074/jbc.M602404200 16597618

[B22] Hammerschmidt S Tillig MP Wolff S Vaerman JP Chhatwal GS Species-specific binding of human secretory component to SpsA protein of Streptococcus pneumoniae via a hexapeptide motif Mol Microbiol 2000 36 3 726 736 10.1046/j.1365-2958.2000.01897.x 10844660

[B23] Bergmann S Rohde M Chhatwal GS Hammerschmidt S alpha-Enolase of Streptococcus pneumoniae is a plasmin(ogen)-binding protein displayed on the bacterial cell surface Mol Microbiol 2001 40 6 1273 1287 10.1046/j.1365-2958.2001.02448.x 11442827

[B24] Bergmann S Rohde M Hammerschmidt S Glyceraldehyde-3-phosphate dehydrogenase of Streptococcus pneumoniae is a surface-displayed plasminogen-binding protein Infect Immun 2004 72 4 2416 2419 10.1128/IAI.72.4.2416-2419.2004 15039372 PMC375162

[B25] Attali C Frolet C Durmort C Offant J Vernet T Di Guilmi AM Streptococcus pneumoniae choline-binding protein E interaction with plasminogen/plasmin stimulates migration across the extracellular matrix Infect Immun 2008 76 2 466 476 10.1128/IAI.01261-07 18070889 PMC2223458

[B26] Attali C Durmort C Vernet T Di Guilmi AM The interaction of Streptococcus pneumoniae with plasmin mediates transmigration across endothelial and epithelial monolayers by intercellular junction cleavage Infect Immun 2008 76 11 5350 5356 10.1128/IAI.00184-08 18725422 PMC2573366

[B27] Schneewind O Model P Fischetti VA Sorting of protein A to the staphylococcal cell wall Cell 1992 70 2 267 281 10.1016/0092-8674(92)90101-H 1638631

[B28] Tettelin H Nelson KE Paulsen IT Eisen JA Read TD Peterson S Heidelberg J DeBoy RT Haft DH Dodson RJ Complete genome sequence of a virulent isolate of Streptococcus pneumoniae Science 2001 293 5529 498 506 10.1126/science.1061217 11463916

[B29] Hoskins J Alborn WE Jr Arnold J Blaszczak LC Burgett S DeHoff BS Estrem ST Fritz L Fu DJ Fuller W Genome of the bacterium Streptococcus pneumoniae strain R6 J Bacteriol 2001 183 19 5709 5717 10.1128/JB.183.19.5709-5717.2001 11544234 PMC95463

[B30] Chhatwal GS Preissner KT Extracellular Matrix Interactions with Gram Positive Pathogens Gram Positive Pathogens, American Society for Microbiology 2000 78 86

[B31] Kostrzynska M Wadstrom T Binding of laminin, type IV collagen, and vitronectin by Streptococcus pneumoniae Zentralbl Bakteriol 1992 277 1 80 83 1381646 10.1016/s0934-8840(11)80874-1

[B32] Tillett WS Francis T Serological reactions in Pneumonia with a non-protein somatic franction of pneumococcus J Exp Med 1930 52 561 571 10.1084/jem.52.4.561 19869788 PMC2131884

[B33] van der Flier M Chhun N Wizemann TM Min J McCarthy JB Tuomanen EI Adherence of Streptococcus pneumoniae to immobilized fibronectin Infect Immun 1995 63 11 4317 4322 7591065 10.1128/iai.63.11.4317-4322.1995 PMC173614

[B34] Bernstein JM Reddy M Bacteria-mucin interaction in the upper aerodigestive tract shows striking heterogeneity: implications in otitis media, rhinosinusitis, and pneumonia Otolaryngol Head Neck Surg 2000 122 4 514 520 10.1016/S0194-5998(00)70093-3 10740170

[B35] Gosink KK Mann ER Guglielmo C Tuomanen EI Masure HR Role of novel choline binding proteins in virulence of Streptococcus pneumoniae Infect Immun 2000 68 10 5690 5695 10.1128/IAI.68.10.5690-5695.2000 10992472 PMC101524

[B36] Molina R Gonzalez A Stelter M Perez-Dorado I Kahn R Morales M Campuzano S Campillo NE Mobashery S Garcia JL Crystal structure of CbpF, a bifunctional choline-binding protein and autolysis regulator from Streptococcus pneumoniae EMBO Rep 2009 10 3 246 251 10.1038/embor.2008.245 19165143 PMC2658566

[B37] Barocchi MA Ries J Zogaj X Hemsley C Albiger B Kanth A Dahlberg S Fernebro J Moschioni M Masignani V A pneumococcal pilus influences virulence and host inflammatory responses Proc Natl Acad Sci USA 2006 103 8 2857 2862 10.1073/pnas.0511017103 16481624 PMC1368962

[B38] Rose L Shivshankar P Hinojosa E Rodriguez A Sanchez CJ Orihuela CJ Antibodies against PsrP, a novel Streptococcus pneumoniae adhesin, block adhesion and protect mice against pneumococcal challenge J Infect Dis 2008 198 3 375 383 10.1086/589775 18507531

[B39] Zipfel PF Hallstrom T Hammerschmidt S Skerka C The complement fitness factor H: role in human diseases and for immune escape of pathogens, like pneumococci Vaccine 2008 26 Suppl 8 I67 74 10.1016/j.vaccine.2008.11.015 19388168

[B40] Dave S Brooks-Walter A Pangburn MK McDaniel LS PspC, a pneumococcal surface protein, binds human factor H Infect Immun 2001 69 5 3435 3437 10.1128/IAI.69.5.3435-3437.2001 11292770 PMC98306

[B41] van Bueren AL Higgins M Wang D Burke RD Boraston AB Identification and structural basis of binding to host lung glycogen by streptococcal virulence factors Nat Struct Mol Biol 2007 14 1 76 84 10.1038/nsmb1187 17187076

[B42] Bethe G Nau R Wellmer A Hakenbeck R Reinert RR Heinz HP Zysk G The cell wall-associated serine protease PrtA: a highly conserved virulence factor of Streptococcus pneumoniae FEMS Microbiol Lett 2001 205 1 99 104 10.1111/j.1574-6968.2001.tb10931.x 11728722

[B43] Thompson D Pepys MB Wood SP The physiological structure of human C-reactive protein and its complex with phosphocholine Structure 1999 7 2 169 177 10.1016/S0969-2126(99)80023-9 10368284

[B44] Aslanidis C de Jong PJ Ligation-independent cloning of PCR products (LIC-PCR) Nucleic Acids Res 1990 18 20 6069 6074 10.1093/nar/18.20.6069 2235490 PMC332407

[B45] Fernandez-Tornero C Lopez R Garcia E Gimenez-Gallego G Romero A A novel solenoid fold in the cell wall anchoring domain of the pneumococcal virulence factor LytA Nat Struct Biol 2001 8 12 1020 1024 10.1038/nsb724 11694890

[B46] Hermoso JA Lagartera L Gonzalez A Stelter M Garcia P Martinez-Ripoll M Garcia JL Menendez M Insights into pneumococcal pathogenesis from the crystal structure of the modular teichoic acid phosphorylcholine esterase Pce Nat Struct Mol Biol 2005 12 6 533 538 10.1038/nsmb940 15895092

[B47] Zhang Z Li W Frolet C Bao R di Guilmi AM Vernet T Chen Y Structure of the choline-binding domain of Spr1274 in Streptococcus pneumoniae Acta Crystallogr Sect F Struct Biol Cryst Commun 2009 65 Pt 8 757 761 10.1107/S1744309109025329 19652332 PMC2720326

[B48] McDaniel LS Scott G Widenhofer K Carroll JM Briles DE Analysis of a surface protein of Streptococcus pneumoniae recognised by protective monoclonal antibodies Microb Pathog 1986 1 6 519 531 10.1016/0882-4010(86)90038-0 3508498

[B49] Talkington DF Crimmins DL Voellinger DC Yother J Briles DE A 43-kilodalton pneumococcal surface protein, PspA: isolation, protective abilities, and structural analysis of the amino-terminal sequence Infect Immun 1991 59 4 1285 1289 2004810 10.1128/iai.59.4.1285-1289.1991 PMC257840

[B50] Garcia JL Sanchez-Beato AR Medrano FJ Lopez R Versatility of choline-binding domain Microb Drug Resist 1998 4 1 25 36 10.1089/mdr.1998.4.25 9533722

[B51] Garcia P Gonzalez MP Garcia E Lopez R Garcia JL LytB, a novel pneumococcal murein hydrolase essential for cell separation Mol Microbiol 1999 31 4 1275 1281 10.1046/j.1365-2958.1999.01238.x 10096093

[B52] Garcia P Paz Gonzalez M Garcia E Garcia JL Lopez R The molecular characterization of the first autolytic lysozyme of Streptococcus pneumoniae reveals evolutionary mobile domains Mol Microbiol 1999 33 1 128 138 10.1046/j.1365-2958.1999.01455.x 10411730

[B53] Garcia P Garcia JL Garcia E Lopez R Nucleotide sequence and expression of the pneumococcal autolysin gene from its own promoter in Escherichia coli Gene 1986 43 3 265 272 10.1016/0378-1119(86)90215-5 2875013

[B54] Sanchez-Beato AR Lopez R Garcia JL Molecular characterization of PcpA: a novel choline-binding protein of Streptococcus pneumoniae FEMS Microbiol Lett 1998 164 1 207 214 10.1016/S0378-1097(98)00206-7 9675866

[B55] Rosenow C Ryan P Weiser JN Johnson S Fontan P Ortqvist A Masure HR Contribution of novel choline-binding proteins to adherence, colonization and immunogenicity of Streptococcus pneumoniae Mol Microbiol 1997 25 5 819 829 10.1111/j.1365-2958.1997.mmi494.x 9364908

[B56] Clarke VA Platt N Butters TD Cloning and expression of the beta-N-acetylglucosaminidase gene from Streptococcus pneumoniae. Generation of truncated enzymes with modified aglycon specificity J Biol Chem 1995 270 15 8805 8814 10.1074/jbc.270.15.8805 7721787

[B57] Oggioni MR Memmi G Maggi T Chiavolini D Iannelli F Pozzi G Pneumococcal zinc metalloproteinase ZmpC cleaves human matrix metalloproteinase 9 and is a virulence factor in experimental pneumonia Mol Microbiol 2003 49 3 795 805 10.1046/j.1365-2958.2003.03596.x 12864860

[B58] Jedrzejas MJ Unveiling molecular mechanisms of bacterial surface proteins: Streptococcus pneumoniae as a model organism for structural studies Cell Mol Life Sci 2007 64 21 2799 2822 10.1007/s00018-007-7125-8 17687514 PMC11136149

[B59] Li S Kelly SJ Lamani E Ferraroni M Jedrzejas MJ Structural basis of hyaluronan degradation by Streptococcus pneumoniae hyaluronate lyase Embo J 2000 19 6 1228 1240 10.1093/emboj/19.6.1228 10716923 PMC305664

[B60] Marion C Limoli DH Bobulsky GS Abraham JL Burnaugh AM King SJ Identification of a pneumococcal glycosidase that modifies O-linked glycans Infect Immun 2009 77 4 1389 1396 10.1128/IAI.01215-08 19139197 PMC2663135

[B61] Abbott DW Macauley MS Vocadlo DJ Boraston AB Streptococcus pneumoniae endohexosaminidase D, structural and mechanistic insight into substrate-assisted catalysis in family 85 glycoside hydrolases J Biol Chem 2009 284 17 11676 11689 10.1074/jbc.M809663200 19181667 PMC2670171

[B62] Zahner D Hakenbeck R The Streptococcus pneumoniae beta-galactosidase is a surface protein J Bacteriol 2000 182 20 5919 5921 10.1128/JB.182.20.5919-5921.2000 11004197 PMC94720

[B63] Novak R Charpentier E Braun JS Park E Murti S Tuomanen E Masure R Extracellular targeting of choline-binding proteins in Streptococcus pneumoniae by a zinc metalloprotease Mol Microbiol 2000 36 2 366 376 10.1046/j.1365-2958.2000.01854.x 10792723

[B64] Pearce BJ Yin YB Masure HR Genetic identification of exported proteins in Streptococcus pneumoniae Mol Microbiol 1993 9 5 1037 1050 10.1111/j.1365-2958.1993.tb01233.x 7934910

[B65] Wani JH Gilbert JV Plaut AG Weiser JN Identification, cloning, and sequencing of the immunoglobulin A1 protease gene of Streptococcus pneumoniae Infect Immun 1996 64 10 3967 3974 8926056 10.1128/iai.64.10.3967-3974.1996 PMC174324

[B66] Bumbaca D Littlejohn JE Nayakanti H Lucas AH Rigden DJ Galperin MY Jedrzejas MJ Genome-based identification and characterization of a putative mucin-binding protein from the surface of Streptococcus pneumoniae Proteins 2007 66 3 547 558 10.1002/prot.21205 17115425

[B67] Paterson GK Nieminen L Jefferies JM Mitchell TJ PclA, a pneumococcal collagen-like protein with selected strain distribution, contributes to adherence and invasion of host cells FEMS Microbiol Lett 2008 285 2 170 176 10.1111/j.1574-6968.2008.01217.x 18557785

[B68] Camara M Boulnois GJ Andrew PW Mitchell TJ A neuraminidase from Streptococcus pneumoniae has the features of a surface protein Infect Immun 1994 62 9 3688 3695 8063384 10.1128/iai.62.9.3688-3695.1994 PMC303019

[B69] Obert C Sublett J Kaushal D Hinojosa E Barton T Tuomanen EI Orihuela CJ Identification of a Candidate Streptococcus pneumoniae core genome and regions of diversity correlated with invasive pneumococcal disease Infect Immun 2006 74 8 4766 4777 10.1128/IAI.00316-06 16861665 PMC1539573

[B70] Yamaguchi M Terao Y Mori Y Hamada S Kawabata S PfbA, a novel plasmin- and fibronectin-binding protein of Streptococcus pneumoniae, contributes to fibronectin-dependent adhesion and antiphagocytosis J Biol Chem 2008 283 52 36272 36279 10.1074/jbc.M807087200 18974092 PMC2662297

